# Inhibition of Proliferation and Induction of Apoptosis in Multiple Myeloma Cell Lines by CD137 Ligand Signaling

**DOI:** 10.1371/journal.pone.0010845

**Published:** 2010-05-26

**Authors:** Charles Gullo, Liang Kai Koh, Wan Lu Pang, Kian Tong Ho, Shi Hao Tan, Herbert Schwarz

**Affiliations:** 1 Cancer Immunology Laboratory, Department of Clinical Research, Singapore General Hospital, Singapore, Singapore; 2 Department of Physiology, Yong Loo Lin School of Medicine, National University of Singapore, Singapore, Singapore; University Paris Sud, France

## Abstract

**Background:**

Multiple myeloma (MM) is a malignancy of terminally-differentiated plasma cells, and the second most prevalent blood cancer. At present there is no cure for MM, and the average prognosis is only three to five years. Current treatments such as chemotherapy are able to prolong a patient's life but rarely prevent relapse of the disease. Even hematopoietic stem cell transplants and novel drug combinations are often not curative, underscoring the need for a continued search for novel therapeutics. CD137 and its ligand are members of the Tumor Necrosis Factor (TNF) receptor and TNF superfamilies, respectively. Since CD137 ligand cross-linking enhances proliferation and survival of healthy B cells we hypothesized that it would also act as a growth stimulus for B cell cancers.

**Methodology/Principal Findings:**

Proliferation and survival of B cell lymphoma cell lines were not affected or slightly enhanced by CD137 ligand agonists *in vitro*. But surprisingly, they had the opposite effects on MM cells, where CD137 ligand signals inhibited proliferation and induced cell death by apoptosis. Furthermore, secretion of the pro-inflammatory cytokines, IL-6 and IL-8 were also enhanced in MM but not in non-MM cell lines in response to CD137 ligand agonists. The secretion of these cytokines in response to CD137 ligand signaling was consistent with the observed activation of the classical NF-κB pathway. We hypothesize that the induction of this pathway results in activation-induced cell death, and that this is the underlying mechanism of CD137-induced MM cell death and growth arrest.

**Conclusions/Significance:**

These data point to a hitherto unrecognized role of CD137 and CD137 ligand in MM cell biology. The selective inhibition of proliferation and induction of cell death in MM cells by CD137 ligand agonists may also warrant a closer evaluation of their therapeutic potential.

## Introduction

Multiple myeloma (MM) is a malignancy of terminally differentiated plasma cells that primarily resides at multiple sites in the bone marrow and is clinically characterized by osteolytic lesions, immunodeficiency, and renal disease. MM represents 20% of all new hematological malignancies, making it the second most prevalent blood cancer [1]. Epidemiologic data indicate both an increasing incidence and an earlier age of onset of the disease. Despite currently available chemotherapeutic treatments and autologous transplantation resulting in an improvement in overall survival of patients, MM still remains incurable and the average prognosis is only three years to five years [2].

Stem cell transplantation can provide long-term remission, but the procedure suffers from a high treatment-related mortality, and the majority of patients relapse [3]. More recently, agents with novel mechanisms of action, such as the proteasome inhibitor, bortezomib and immunomodulatory drugs like thalidomide and lenalidomide have shown promise for treatment of patients with refractory and relapsed disease, and for those with previously untreated multiple myeloma [4], [5]. Even with these novel drugs, the majority of MM patients eventually experience relapse, their disease becomes chemoresistant, and they die of the disease [6], [7]. Therefore, an approach that allows targeting and selective killing of cancerous MM cells remains highly desirable.

CD137 (TNFRSF9, 4-1BB, ILA) is a cytokine receptor and a member of the tumor necrosis factor receptor family, and a potent T cell costimulatory molecule [8]–[10]. The ligand for CD137 is expressed by antigen presenting cells (APC) and APC use the CD137 receptor/ligand system to costimulate T cell activity. APC express CD137 ligand as a cell surface transmembrane protein, and CD137 ligand can transduce signals into APC, a process known as reverse signaling. Therefore, bidirectional signaling exist for the CD137 receptor/ligand system [11]. CD137 ligand signals induce activation, survival, proliferation and migration of monocytes [12]–[17], maturation of dendritic cells [18], [19], and proliferation and differentiation of hematopoietic progenitor cells [20], [21]. However, the signaling pathways emanating from the ligand and its physiologic role in immune regulation have only partly been characterized.

Previous data highlights the importance of the CD137 ligand signaling in B cell maturation and activation. In B cells CD137 ligand signaling enhances proliferation and immunoglobulin synthesis [22]. B cells likely receive signals via CD137 ligand when interacting with either CD137-expressing helper T cells or CD137-expressing follicular dendritic cells in germinal centers [22], [23]. It was postulated that similarly to the CD40 receptor/ligand system, which mediates T cell help to B cells after first antigen encounter, the CD137 receptor/ligand system may mediate costimulation of B cells by follicular dendritic cells during affinity maturation [22].

We hypothesized that the CD137 ligand signal may have similar activating effects on malignant B cells, and thereby possibly support B cell cancers. Here we describe that the opposite is true in MM cells where CD137 ligand signals inhibit proliferation and induce cell death by apoptosis, while proliferation and survival of non-MM B cell lymphoma cell lines are not affected. Secretion of the pro-inflammatory cytokines IL-6 and IL-8 is also increased in MM cell lines following CD137 ligand activation but not in non-MM cell lines. CD137 ligand signals also activate the classical NF-κB pathway in the MM cell lines. This data suggests that crosslinking of CD137 ligand on MM cell lines might represent a novel method to specifically target MM cells for destruction. It also suggests that at least in some B cell cancers, CD137 ligand induction results in the early activation of the NF-κB pathway that induces a pro-inflammatory as well as a pro-apoptotic state.

## Materials and Methods

### Recombinant proteins and antibodies

CD137-Fc protein was purified from supernatants of stably transfected CHO cells by protein G sepharose, as described previously [24]. Human IgG1 Fc fragment was purchased from Chemicon (Temecula, CA, USA). Antibodies used in this study includes PE-conjugated mouse IgG1, κ isotype control (clone MOPC-21, Sigma-Aldrich, USA), anti-human CD137 (clone 4B4-1, BD Pharmingen, USA), anti-human 4-1BB ligand (clone 5F4, Biolegend, USA, and clone 41B436 Alexis Biochemicals, Switzerland), unlabelled mouse IgG1, κ isotype control (clone MOPC-21, Sigma, USA).

### Cells

RPMI-8226 and Raji were obtained from ATCC (Manassas, VA, USA). DOHH-2 and SUDHL-4 were obtained from the Deutsche Sammlung von Mikroorganismen und Zellkulturen GmbH (DSMZ, Germany). The SGH-MM5 and SHG-MM6 human MM cell lines were developed in our laboratory from a patient with MM using a modified Dexter-type long-term tissue culture system, as described previously [25].

### Crosslinking of CD137 ligand

CD137 ligand was crosslinked by monoclonal antibodies specific for CD137 ligand, or by a recombinant fusion protein consisting of the extracellular domain of CD137 and the constant domain (Fc) of IgG1 (CD137-Fc). Isotype antibodies or a recombinant Fc protein were used as negative controls, respectively. These proteins were immobilized onto tissue culture plates by coating at 4°C overnight, to enable them to crosslink CD137 ligand on the lymphoma and MM cells. Unless otherwise indicated at 10 µg/ml protein solutions were used.

### Flow cytometric analysis

Aliquots of cultured cells (2−3×10^5^ cells) were stained with respective fluorochrome conjugated antibodies in PBS containing 0.5% FBS and 0.1% sodium azide (FACS buffer) for 1 h at 4°C in the dark. Cells were then washed twice with FACS buffer and resuspended in 500 µl of FACS buffer. Flow cytometry was performed on a FACSort (Becton Dickinson, San Jose, CA) with CellQuest (Becton Dickinson) data acquisition and analysis software. Nonspecific staining was controlled by isotype matched antibodies.

Cell cycle analysis: Cells were resuspended in 200 µl 7-AAD binding buffer (BD Pharmingen). 1.8 ml of ice-cold 70% ethanol were added drop-wise while vortexing, incubated on ice, spun down and resuspended in 150 µl binding buffer. 5 µl of 7-AAD (BD Pharmingen) were added and the cells were left for 15 min in the dark at RT. The volume was adjusted to 400 µl before flow cytometry. Data analysis was performed using the software ModFit.

### Death and apoptosis assays

Live and dead cell counts were performed with a haemocytometer after staining with Trypan Blue (Sigma-Aldrich, USA). Apoptotic and necrotic cells were stained by 10 µg/ml Ethidium Bromide and 3 µg/ml Acridine Orange, and viewed under the IX81 microscope (Olympus, USA). Annexin V externalisation was detected using the Annexin-V Apoptosis Detection Kit (BD Pharmingen, USA), and analyzed by flow cytometry (CyAn™, DakoCytomation, Denmark) and Summit software. Caspase 3 activity was measured using the Caspase 3 Colorimetric Assay Kit [CPP32], (Chemicon).

### Detection of mitochondrial transmembrane potential

3, 3′ dihexyloxacarbocyanine iodide (DiOC_6_), (Invitrogen, San Diego, USA) was used to measure the mitochondrial transmembrane potential Δψ_m_. 10^5^ cells were cultured for 24 h at 37°C in 12-well plates that had been coated with Fc or CD137-Fc protein. Cells were loaded with 50 nM of DIOC_6_ for 30 min in the dark at 37°C. As a positive control, cells were treated with 100 µm H_2_O_2_ for 4 h and then loaded with DIOC_6_. The cells were then harvested, washed and resuspened in 1X PBS and analyzed immediately by flow cytometry as described.

### Proliferation assays

Cells were pulsed with 0.5 µCi of ^3^H-thymidine (PerkinElmer, Boston, MA, USA) for the last 24 h of the culture period. The cells were then harvested onto a Packard Unifilter Plate using a MicroMate 196 Cell Harvester and counted using a TopCount (Perkin Elmer, Waltham, MA, USA).

### ELISA

Cytokine concentrations in cell supernatants were determined by human DuoSet ELISA Development kits (R&D Systems, Minneapolis, MN, USA), according to the manufacturer's instructions. All measurements were performed in triplicate.

### Preparation of cell lysates

1.2×10^7^ cells were starved of serum, treated under the indicated conditions and collected for cell lysis to obtain protein samples. Cells were lysed with EBC lysis buffer (1 M Tris-HCL pH 8.0, 1.5 M NaCl, NP-40, 0.5 M NaF, 100 mM Na_3_VO_4_, 250 mg/ml PMSF, dH_2_0) (Roche Diagnostics, GmbH, Mannheim, Germany). Complete Mini Protease Inhibitor Cocktail Tablets, (Sigma Aldrich, St. Louis, MO, USA) and Phosphatase Inhibitor Cocktails 1 and 2 (Santa Cruz Biotechnology, Santa Cruz, CA, USA) were added to prevent protein digestion and dephosphorylation, respectively. Cell lysates were then spun at 14,000 rpm for 10 min to obtain supernatants. Protein samples were stored at −80°C when not used. Protein concentrations were determined using the Bio-Rad Bradford assay (Bio-Rad Laboratories, Hercules, CA, USA).

### Immunblotting

Protein samples were resolved with 10% SDS Polyacrylamide gel. 80 µg of protein sample were loaded in each well with 4x sample buffer. Protein samples were then resolved by electrophoresis (120 V two hours). The resolved proteins were next transferred from the polyacrylamide gel to Millipore Immobilon-P^SQ^ Transfer PVDF membranes (Millipore, Billerica, MA, USA) using the Bio-Rad SD Semi-dry Transfer system (5 V overnight at 4°C). Membranes were then blocked with a solution of 5% non-fat milk, 1% Tween 20 in Tris Buffered Saline. Next, the membranes were incubated with various primary antibodies (Cell Signaling Technology, Inc, Beverly, MA, USA) at 4°C overnight, washed and detected using HRP-conjugated secondary antibodies and Thermo Pierce (Rockford, IL, USA) SuperSignal® West Pico Chemiluminescent Substrates. Images of the western blots were visualized and recorded using the Alpha-Innotech FluorChem® (Alpha Innotech, San Leandro, CA, USA) system.

### RNA extraction

Total RNA was extracted from 10^6^ cultured cells with the appropriate conditions using Rneasy Mini Kit (QIAGEN, Valencia, CA), according to manufacturer's manual. Concentration and purity of the RNA extracted was determined by spectrophotometry using a 1∶10 diluted sample.

### Real time reverse transcription PCR

Gene sequences for IL-6 and IκBα were obtained from GeneBank. Primers for target gene sequences were designed using Roche Universal Probe Library Assay Design Centre (http://www.universalprobelibrary.com). The primers used were as follows: *IL-6*: 5′ – cag gag ccc agc tat gaa ct – 3′ (forward) and 5′ – agc agg caa cac cag gag – 3′ (reverse), for *IκBα*: 5′ – gac gag gag tac gag cag atg – 3′ (forward) and 5′ – atg gcc aag tgc agg aac – 3′ (reverse) and *GAPDH*: 5′ – gag tcc act ggc gtc ttc ac – 3′ (forward) and 3′ – ttc aca ccc atg acg aac at – 3′ (reverse). One step Real Time Reverse Transcription PCR (RT-PCR) was performed using Roche LightCycler® system (Roche Diagnostics, GmbH, Mannheim, Germany). A calibrator control and GAPDH control were included in every analysis for comparison. The relative fold change for each gene was calculated using 2^−ΔΔCT^ method. The ΔΔCT formula used for establishing fold change is as follows: ΔΔCT  =  (Cp _target gene_ – Cp _GAPDH_) – (Cp _target gene_ – Cp _control_).

### Nuclear extraction

Nuclear proteins were extracted and isolated from multiple myeloma cells using the Thermo Scientific NE-PER® Nuclear and Cytoplasmic Extraction Kit protocol. The cells were lysed in cytoplasm extraction reagent and spun at 14,000 g to extract the nuclear material. Proteins from the nuclear material were then extracted by adding nuclear extraction reagent to the nuclei and spun at 14,000 g. Nuclear extracts were stored at −80°C until used. Protein concentrations of the nuclear extracts were measured using Bio-Rad Bradford protein quantification assay.

### NF-κB family transcription factor colorimetric assay

The levels of NF-κB transcription factors (p50, p65, p52 and RelB) present in the nuclei of treated cells were detected using the Active Motif (Carlsbad, CA, USA) TransAM™ NF-κB Family Transcription Factor Assay Kit. Absorbance of individual wells were measured at 450 nm for 0.1 seconds using the Victor3™ spectrophotomer (Perkin Elmer, Waltham, MA).

### Statistics

Statistical significance was determined using a two-tailed Student's t-test.

## Results

### B cell lymphoma cells express CD137 ligand but not of CD137

**Figure 1:**
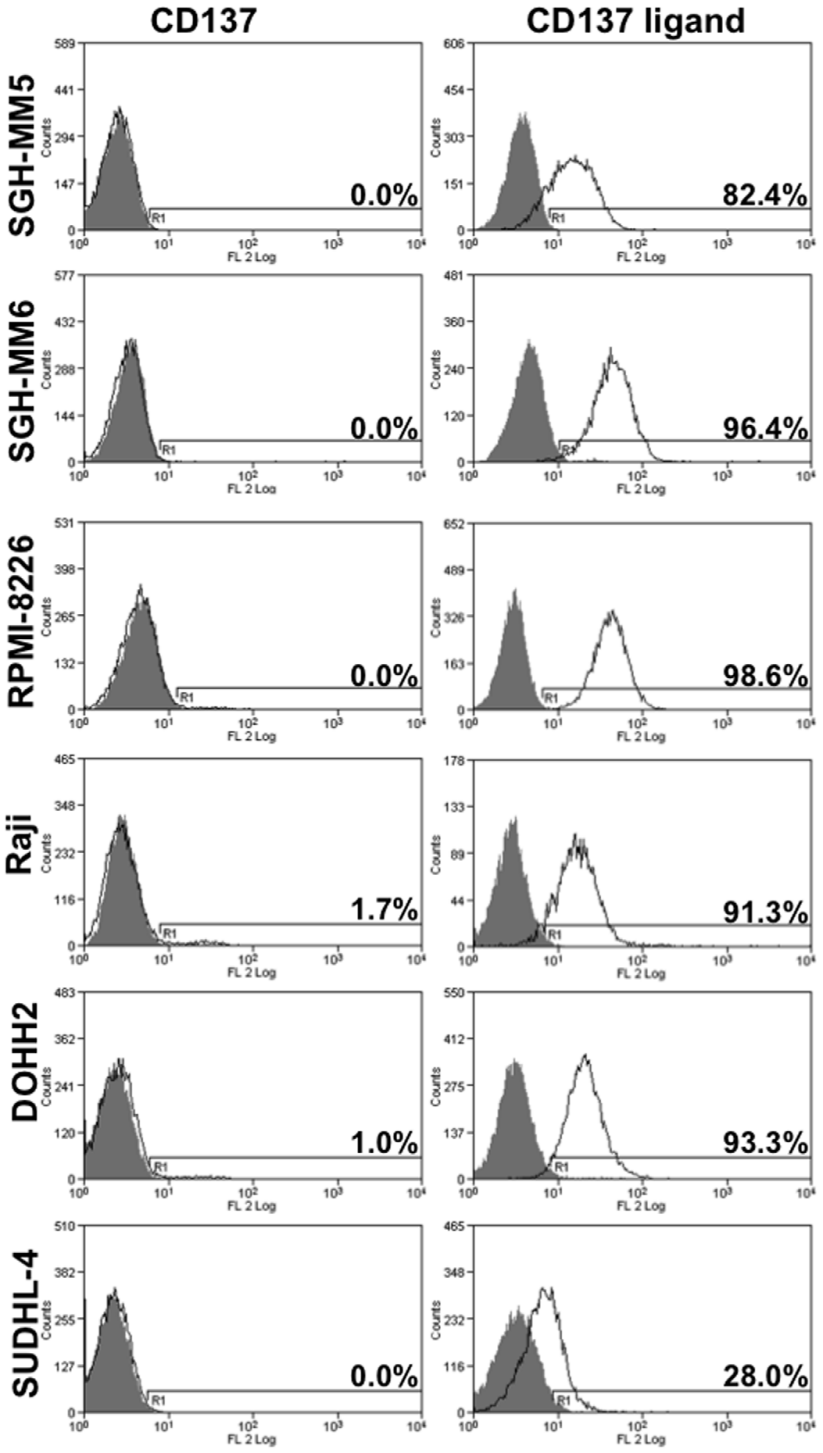
CD137 ligand is expressed by B cell lymphoma and myeloma cell lines. Cells were stained by PE-conjugated monoclonal antibodies against CD137 (clone 4B4-1), or anti-CD137 ligand (clone 4B1-436), (open curves) or their isotype control (MOPC-21), (filled curve).

The constitutive expression of CD137 ligand by primary B cells provides the molecular basis for B cells to receive costimulatory signals from CD137 [26], [27]. Therefore, as a first step in investigating the effects of CD137 on B cell lymphoma cell lines we tested CD137 ligand expression. For our studies we selected the Burkitt's lymphoma Raji, the non Hodgkin lymphoma SUDHL-4, the B cell lymphoma DOHH-2 and the three multiple myeloma (MM) lines SGH-MM5, SGH-MM6 and RPMI 8226. All six cell lines express CD137 ligand constitutively, but none expresses CD137, a situation identical to that of primary B cells (Figure 1).

### CD137 inhibits proliferation of MM cells

**Figure 2:**
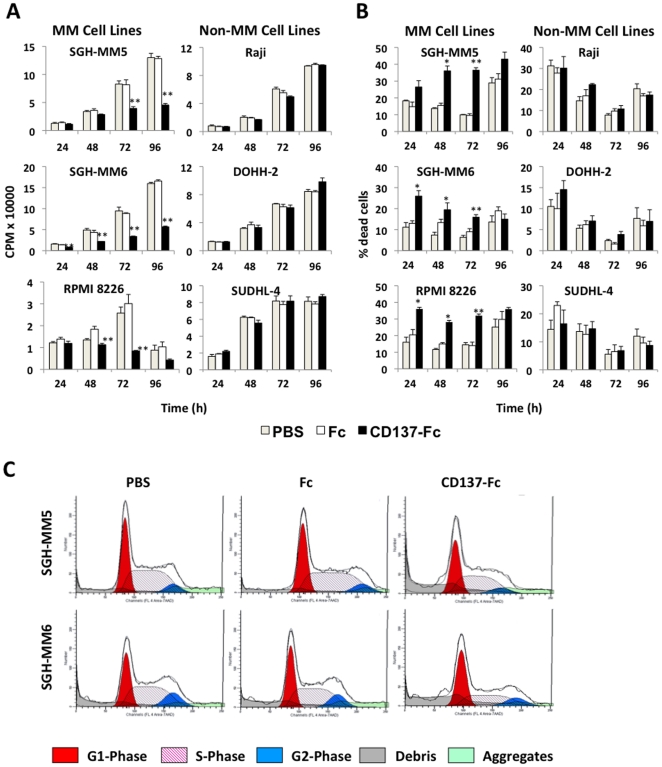
CD137 inhibits proliferation and induces cell death of MM but not of non-MM cells. Cells were cultured on plate-bound Fc or CD137-Fc protein or on uncoated plates (PBS). (A) After indicated times proliferation was determined via ^3^H-thymidine incorporation. (B) Cell viability was determined after 24, 48, 72 and 96 h via trypan blue staining. Depicted are means ± standard deviations of triplicate measurements. * p<0.05. This experiment is representative of three independent experiments with similar results.

Since CD137 ligand crosslinking enhances proliferation of preactivated B cells, we tested this activity in B cell lines [22], [28]. CD137 ligand stimulation had no significant effect on the proliferation of the Raji, DOHH-2 and SUDHL-4 cells over three days as assessed by ^3^H-thymidine incorporation (Figure 2A). In contrast, proliferation of the three MM cell lines SGH-MM5, SGH-MM6 and RPMI 8226 was profoundly decreased by CD137. This inhibitory effect was most visible at the later time point of 72 h (Figure 2A). Titration of the CD137-Fc protein revealed that inhibition of proliferation was of comparable magnitude between 2.5 and 20 µg/ml, indicating that at 10 µg/ml CD137 protein is already at its saturation point.

### CD137 induces cell death in the MM cell lines by apoptosis

In order to investigate the mechanism behind the inhibition of proliferation, we asked next whether CD137 ligand ligation on MM cells arrested cell cycle progression or induced cell death. The percentage of dead cells was increased up to 2 to 3-fold in MM cells after 6 or 24 hours of culture on CD137-Fc compared to Fc protein (Figure 2B). Viability of the non-MM B cell lymphoma (non-MM) cell lines was not affected by CD137 ligand signaling.

Cell cycle analysis using 7-AAD staining on SHG-MM5 and SGH-MM6 cell confirmed induction of MM cell death by CD137 ligand signaling as evidenced by the increase in hypodiploid DNA (sub-G1/debris peak), (Figure 2C). There was also a decrease in the number of cells in the S phase, indicating that in addition to induction of apoptosis cell cycle arrest also contributes to the inhibitory effect of CD137 ligand signaling.

**Figure 3:**
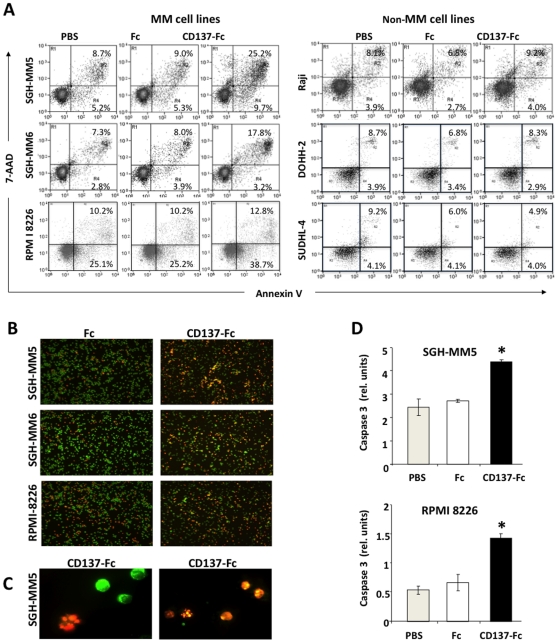
CD137 induces apoptosis in the MM cell lines. (A) SGH-MM5 cells at a density of 10^6^ cells/ml were cultured on plate-bound Fc or CD137-Fc protein or on uncoated plates (PBS). After 24 h the cells were stained with Annexin V and 7-AAD. Similar results were obtained for the other MM cell lines. (B) Cells from (A) were stained with Acridine Orange (green) and Ethidium Bromide (red). Photographs were taken at a magnification of 40×. (C) CD137-Fc treated SGH-MM5 cells of B at a magnification of 200×. (D) Caspase 3 activity was determined 6 h after exposure of SGH-MM5 and RPMI 8226 cells to immobilized Fc or CD137-Fc protein. These experiments are representative of three independent experiments with similar results.

We next asked whether this reduction in viability was due to CD137-Fc induced MM cell apoptosis. Annexin-V and 7-AAD staining revealed increases in the percentages of early (Annexin V^+^, 7-AAD^−^) and late (Annexin V^+^, 7-AAD^+^) apoptotic cells at 24 hours (Figure 3A). Consistent with the results from the proliferation and viability assays (Figure 2), apoptosis rates of non-MM cell lines were not affected. Induction of apoptosis was further confirmed by ethidium bromide and acridine orange staining which showed extensive chromatin condensation and membrane blebbing after treatment with CD137-Fc (Figure 3B,C) and by activation of caspase 3 (Figure 3D). Thus, CD137 reverse signaling results in an arrest of proliferation as well as an induction of apoptosis in MM cells while it has no effect on either parameter in non MM cell lines.

### Requirement of immobilisation of CD137 ligand agonists

**Figure 4:**
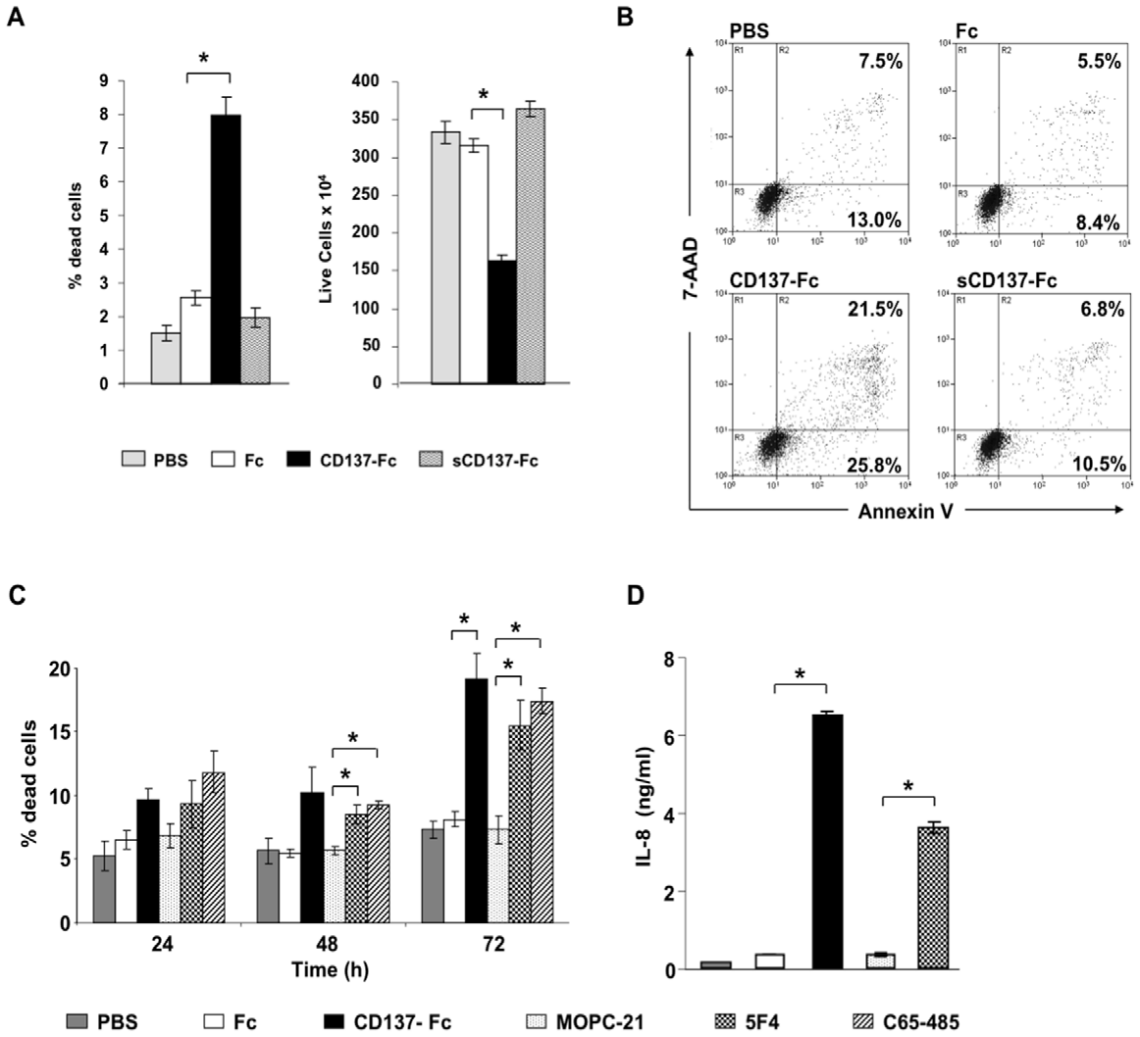
Requirement of immobilization of CD137 ligand agonists. SGH-MM5 (A and B) or SGH-MM6 (C and D) cells at a density of 10^6^ cells/ml were cultured on uncoated plates (PBS), or on plate-bound Fc, CD137-Fc, mouse IgG (MOPC21) or anti-CD137 ligand antibody (clones 5F4 and C65-485) or on uncoated plates (PBS), or to which Fc or CD137-Fc proteins were added soluble at 10 µg/ml. (A) Percentage of dead cells (left panel) and number of total live cells (right panel) were determined after 24 h via trypan blue staining. (B) Extent of apoptosis of cells in (A) was determined by Annexin V and 7-AAD staining. (C) Percentages of dead cells were determined at indicated times via trypan blue staining. (D) IL-8 concentrations in 24 h cell supernatants as determined by ELISA. Depicted are means ± standard deviations of triplicate measurements. * p<0.05.

Many TNF receptor family members such as CD95 require trimerization and higher order multimerization to initiate signaling [29]. We observed that cross-linking of CD137 ligand was essential for induction of cell death and for the reduction of live cell numbers since addition of recombinant CD137-Fc protein in a soluble form had no effect (Figure 4A). Also, numbers of live and apoptotic cells were not different between uncoated wells (PBS) and Fc protein-coated wells demonstrating no influence of the Fc control protein (Figure 4B). In the experiments above, recombinant CD137 protein was used to crosslink CD137 ligand on MM cells. Anti-CD137 ligand antibodies which can also crosslink CD137 ligand had the same functional effects on MM cell lines. The two monoclonal anti-CD137 ligand antibodies, clones 5F4 and C65-485, induced cell death (Figure 4C), and cytokine secretion (Figure 4D) in MM cells to a similar extent as the recombinant CD137-Fc protein. Thus, similar to forward signaling through receptors of the TNF receptor family, reverse signaling through CD137 ligand also requires oligomerization which is consistent with previous studies [24].

### Engagement of MM cells via CD137 results in the expression pro-inflammatory cytokines

Cytokines crucially influence proliferation, survival and death of healthy and malignant B cell lymphoma cells as well as MM cells. In particular, IL-6 and IL-8 are important growth and survival factors for MM cells. The production of IL-6 and IL-8 is enhanced upon interaction of MM cells with bone marrow stromal cells, and is dependent on NF-κB activation [30]–[32]. VEGF (also highly dependent on NF-κB activity) is crucial in regulating angiogenesis, while transforming growth factor (TGF)-β is often secreted by tumor cells to blunt an anti-tumor immune response, or to increase the cells' threshold for apoptosis induction [33], [34]. In order to understand what soluble factors may be responsible for the observed differences to CD137 ligand signaling between the B cells and MM cells, we next investigated which cytokines if any were expressed.

**Figure 5:**
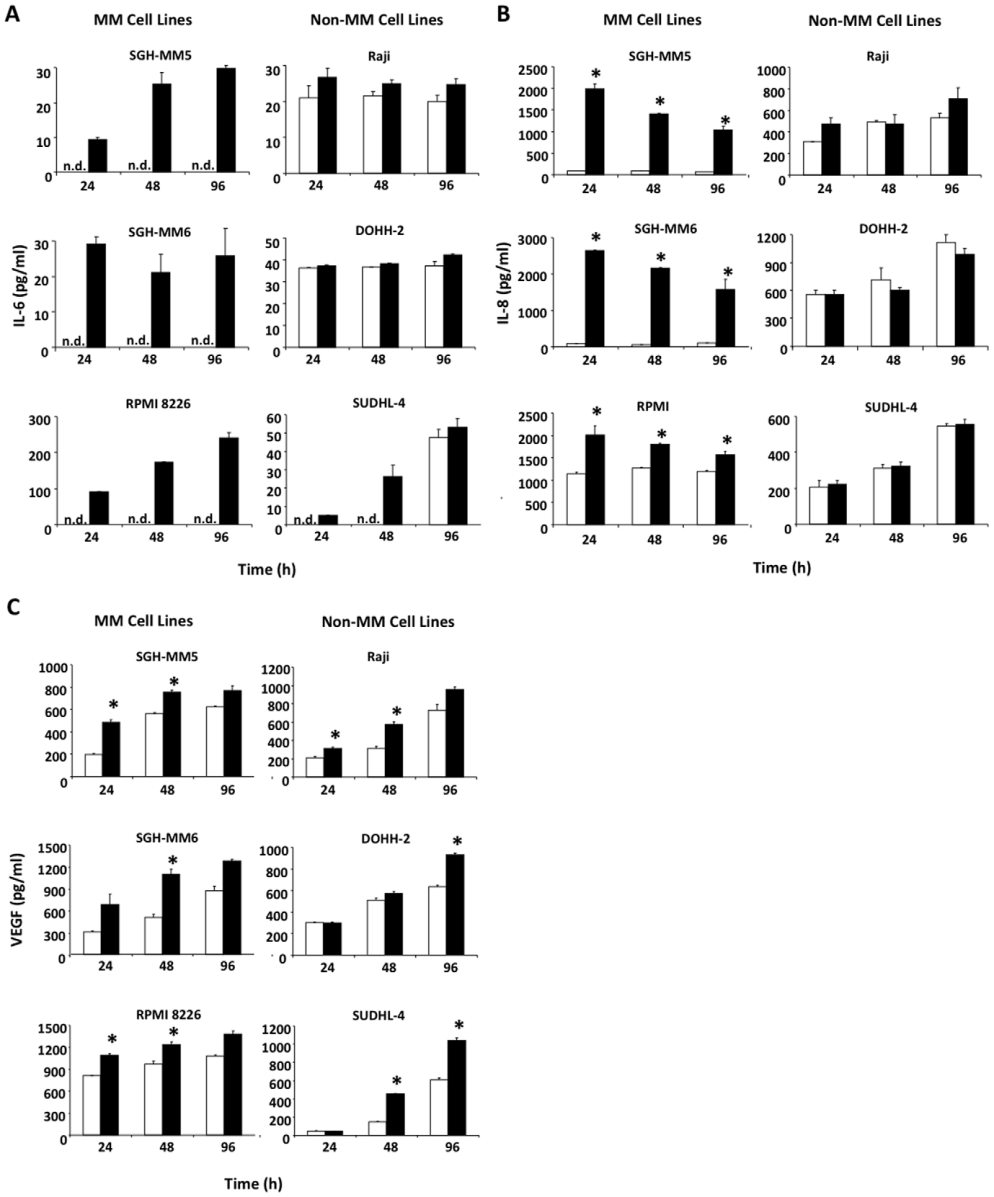
CD137 ligand signaling results in upregulation of pro-inflammatory cytokines in MM but not in non-MM cell lines. Cells at a density of 10^6^ cells/ml were cultured on plate-bound Fc (white bars) or CD137-Fc protein (black bars). (A) IL-6, (B) IL-8 and (C) VEGF concentrations in 24, 48 and 72 h cell supernatants were determined by ELISA. Depicted are means ± standard deviations. * p<0.05. This experiment was performed three times with similar results.

Surprisingly, CD137 ligand agonists induced a strong expression of both IL-6 and IL-8 after 24, 48 or 96 hours that was not observed in the non-MM cell lines (Figure 5A and B). With the exception of IL-8 in RPMI 8226 cells, levels of these two cytokines were either below the detection limit or produced in negligible amounts in the control unstimulated conditions. VEGF secretion was enhanced moderately in both MM and non-MM cell lines by CD137-Fc (Figure 5C), whereas levels of TGF-β were not affected (not shown). Thus, like normal B cells, MM B cells respond by producing pro-inflammatory/pro-survival cytokines despite the observed induction of apoptosis and arrest of growth in MM cells.

### Survival signals do not prevent CD137-induced apoptosis of MM cells

**Figure 6:**
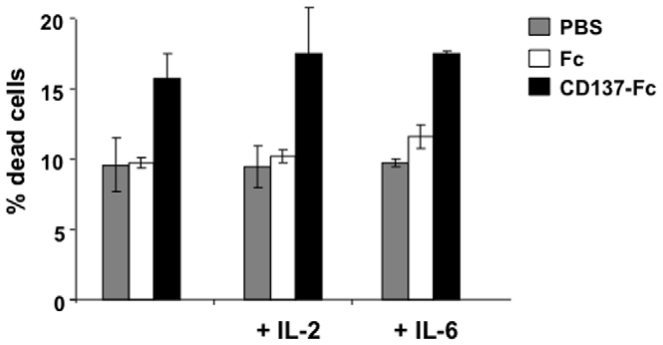
CD137-induced MM cell death is not inhibited by IL-6 or IL-2. SGH-MM6 cells at a density of 1.2×10^6^ cells/ml were cultured on plate-bound Fc or CD137-Fc protein or on uncoated plates (PBS), and IL-6 (1 ng/ml) or IL-2 (100 units/ml) were added. Cell viability was determined after 24 h via trypan blue staining. Depicted are means ± standard deviations of percentages live cells from triplicate measurements. * p<0.05. This experiment is representative of three independent experiments with similar results.

It was surprising to discover that CD137-induced secretion of IL-6, a potent survival factor for MM cells, given its simultaneous suppression of proliferation and induction of apoptosis in MM cell lines. Therefore, we tested whether IL-6 interferes with CD137 ligand-induced cell death. We also included IL-2, the classical lymphocyte growth and survival factor. Apoptosis was induced in MM cells by immobilized CD137-Fc protein in the presence of IL-6 or IL-2. Neither cytokine could rescue MM cells from CD137-induced apoptosis (Figure 6). In addition, blocking the IL-6 receptor by neutralizing antibodies had no effect on CD137-induced apoptosis in MM cells (data not shown). Thus, the pro-apoptotic and growth arrest properties of CD137 reverse signaling appear to be stronger than the survival response via the production of IL-6, IL-8 and VEGF.

### CD137-induced apoptosis of MM cells occurs through activation induced cell death

**Figure 7:**
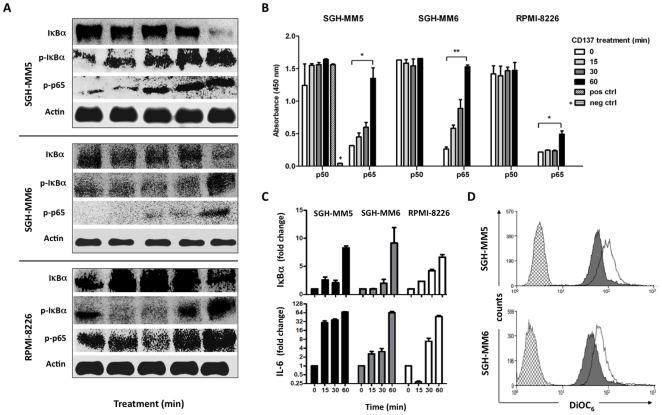
Early activation of the classical NF-κB pathway is initiated upon CD137 ligand signaling. SGH-MM5, SGH-MM6 and RPMI 8226 cells were treated with plate-bound Fc or CD137-Fc. (A) Total protein was extracted at indicated times. NF-κB signaling proteins were detected by immunoblotting. (B) Cells were treated with CD137-Fc for indicated times and nuclear extracts were isolated and subjected to NF-κB transcription factor assay analysis. Data is represented by the average of triplicates within the experiment and is representative of two independent experiments. P values were calculated using pair wise t-test comparing time zero to time 60 min. * p<0.05. (C) Cells were treated with plate bound Fc or CD137-Fc protein for six hours following which total RNA was extracted. Real time RT-PCR was performed on both human IκBα and IL-6 transcripts. RT-PCR data is represented by fold change as calculated using the 2^ΔΔCT^ method where GADPH served as a reference gene, and where each data point was performed in triplicate. (D) Cells were cultured for 48 h on plates with immobilized Fc (white open curve) or CD137-Fc protein (grey filled curve), and then stained with 50 nM DiOC_6_ and analyzed by flow cytometry. Cells with no DiOC_6_ added were used as background control (hatched).

Reverse signaling through CD137 ligand appears to result in two opposing actions, initiation of pro-survival and/or pro-inflammatory pathways and initiation of apoptosis and growth arrest. In order to explain these seemingly contradicting results we investigated signaling pathways that might explain both phenomena. Consecutive induction of cellular activation and cell death is a well known phenomenon in leukocytes and termed activation induced cell death (AICD) [35]. To verify cellular activation in MM cells that could explain the production of the pro-survival cytokines, we tested whether CD137 ligand signaling induces the NF-κB pathway. CD137 stimulation of all three MM cell lines resulted in a reduction of the levels of the inhibitor of NF-κB (IκBα) as well as the phosphorylation of the inhibitor at 60 minutes (Figure 7A). Furthermore, the phosphorylation of p65 (a classical NF- κB transcription factor) was induced in all three cells lines one hour after stimulation by CD137 protein. Phosphorylation of p65 resulted in its nuclear translocation in all three cell lines (Figure 7B). There was no change in the levels of activated p50 as its constitutive levels were already very high (Figure 7B). However, the activity of NF-κB is determined primarily by the p65 subunit because this rate-limiting subunit contains the transcription activation domain [36]. Thus, CD137 ligand signaling resulted in an early induction of the classical NF-κB transcription factor in MM cells in a time period consistent with activation that leads to apoptosis and cytokine production. CD137 induced NF-κB signaling was functional since it led to the increase in the transcription of classical NF-κB-regulated gene products such as IκBα and IL-6 as determined by real-time quantitative RT-PCR (Figure 7C). Thus, this data indicates that CD137 stimulation of MM cells results in a potent and early activation of the classical NF-κB pathway.

AICD in T cells is induced by T cell receptor stimulation, and relies on CD95/CD95 ligand interaction. AICD can be induced in different types of B cells through B cell receptor signaling, and this death induction increases expression of bax, suggesting an involvement of the intrinsic pathway [37] [38]. In addition, AICD induced through B cell receptor signaling or CD40 signaling is independent of CD95 ligand [38], [39]. Indeed, we could not detect any CD137-induced changes CD95 or CD95 ligand, nor in the expression of death receptor (DR)4 and DR5 and TRAIL (not shown). However, CD137 ligand signaling led to mitochondrial membrane depolarization, a characteristic and essential event for apoptosis mediated by the intrinsic pathway [40]. Mitochondrial membrane integrity was assessed by staining with DiOC_6_ whose mean flourescence intensity was reduced by CD137 ligand signaling from 131.1 to 70.5 in SGH-MM5 cells, and from 89.4 to 51.2 in SGH-MM6 cells (Figure 7D). This data suggests that the apoptosis induced by CD137 ligand signals initiate intrinsic apoptotsis pathways.

## Discussion

In this study we identified unexpected activities of CD137 ligand crosslinking on MM cell lines. CD137 ligand signaling inhibited proliferation, induced cell cycle arrest and apoptosis and resulted in an increased secretion of IL-6 and IL-8 selectively in MM cell lines but not in non-MM B cell lymphoma cells. These data were unexpected as CD137 is known to enhance activation and proliferation of primary B cells [22], [28]. We had hypothesized that CD137 would also enhance proliferation of B cell lines, especially since CD137 can be expressed as a neoantigen by certain B cell lymphomas (our unpublished data). In theory, the ectopic expression of CD137 could enable malignant B cells to send and receive growth signals in an auto- or paracrine manner which under physiological conditions are delivered by CD137-expressing helper T cells or follicular dendritic cells [22], [23].

CD137 ligand signals induced secretion of IL-6 and IL-8 specifically in the MM cell lines but not the non MM B cell lymphoma lines. IL-6 has been shown to be essential for MM growth and for protection from apoptosis [31], [32], and increased IL-6 levels in MM patient sera correlate with disease progression [33], [41]. Similarly, IL-8 supports MM growth, and IL-8 secretion by bone marrow stromal also correlates with MM progression [30]. VEGF is also induced by CD137 ligand signals although to a lower extent, and in both MM and non-MM cell lines. VEGF is a potent growth factor for MM cells, and supports MM growth by inducing angiogenesis [42], [43]. All of these cytokines are strongly induced at the transcriptional level by the classical NF-κB pathways that rely on the activation of transcription factors, p65/p50.

It is at first sight surprising that a cellular signal can induce both cell death and cell activation at the same time. NF-κB activation and secretion of pro-survival cytokines clearly indicate a role for cellular activation by CD137 ligand signaling. Induction of apoptosis could be a result of AICD. AICD is a well known phenomenon in activated lymphocytes [35]. The finding that CD137 ligand agonist-induced apoptosis is not inhibited by addition of IL-6 or IL-2 also supports AICD as a potential mechanism of CD137 ligand-induced apoptosis.

CD40 shares many similarities with CD137, and indeed anti-CD40 antibodies have a direct cytotoxic effect in various B cell malignancies including MM [44], [45]. Phase I clinical trials with CD40 agonists are being conducted on MM patients with encouraging results [46]. Although the exact mechanism of anti-CD40 induced B cell death is not known, the most plausible mechanism is one that relies on AICD [47], [48]. However, CD40 is also known to exert proliferative effects as well. For example, soluble CD40 ligand is sufficient to induce proliferation of MM cells whereas normal B cells require both CD40 ligand and IL-4 for full activation [25], suggesting that CD40 ligand act differently on different cells, and that MM cells exist at a stage of differentiation or activation that does not require a second signal such as IL-4. Finally, CD40 stimulation has been shown to induce apoptosis or proliferation in different cell types despite the activation of the NF-κB pathway in both instances [46]. Thus, just as CD40 signaling results in proliferation in some cells and apoptosis in others, CD137 ligand signaling may have similar diverse effects depending on the cell type.

Non-MM cell lines expressed similar levels of CD137 ligand as the MM cell lines. And crosslinking of CD137 ligand enhanced VEGF secretion in non-MM cell lines demonstrating that CD137 ligand signaling is functional in these cells. However, inhibition of proliferation, induction of apoptosis and IL-6 and IL-8 secretion were specific for the MM cell lines. The molecular basis of this difference in biological responses between MM and non-MM cell lines is not known but is currently being addressed by ongoing research. One possible explanation could be provided by the recent finding that many MM tumors have constitutively activated NF-κB [49], [50]. Therefore, any additional stimulation such as by CD137 or by anti-CD40 antibodies may induce AICD in MM cells. It will be interesting to determine the exact intracellular mechanisms of the proposed AICD and compare the components of that system between B lymphoma and MM cells.

An important question arising from this data is whether CD137 ligand agonists can also induce death of malignant plasma cells from MM patients. If that proves to be the case a recombinant CD137 protein or anti-CD137 ligand antibodies could be evaluated for MM therapy. It would be especially important to assess whether CD137 ligand agonists amplify the therapeutic effects and synergize with current MM therapies such as dexamethasone, thalidomide or proteasome inhibitors.
